# Cervical cancer screening among women with comorbidities: evidence from the 2022 Tanzania demographic and health survey

**DOI:** 10.1186/s12889-024-18552-4

**Published:** 2024-04-19

**Authors:** Joshua Okyere, Castro Ayebeng, Abigail Kabukie Dosoo, Kwamena Sekyi Dickson

**Affiliations:** 1 Department of Population and Health, University of Cape Coast, Cape Coast, Ghana; 2 Department of Nursing, College of Health Sciences, Kwame Nkrumah University of Science and Technology, Kumasi, Ghana; 3 Department of Human Resource Management, School of Business, University of Cape Coast, Cape Coast, Ghana

**Keywords:** Cervical cancer, Chronic morbidity, Screening, Hypertension, HIV

## Abstract

**Background:**

The aim of this study is to examine cervical cancer screening (CCS) uptake among women living with hypertension and HIV in Tanzania.

**Methods:**

We used the recently released 2022 Tanzania Demographic and Health Survey. The outcome variable assessed in the study was CCS, whereas chronic morbidities constituted the main explanatory variable. Data analysis was based on observations from 6,298 women aged 30–49 years. Multivariable logistic regression models were used to determine the association between hypertension and HIV status, and CCS uptake. The analyses were computed in STATA 18.

**Results:**

Out of the 6,298 respondents, only 805 (12.8%) had undergone CCS with higher screening uptake among those living with either one of the disease (28.5%) than among those living with neither hypertension or HIV. The highest proportion was found among those who had ever been diagnosed with hypertension (24.1%) and among women with positive HIV test results (36.7%). There was a significantly higher likelihood of undergoing screening for cervical cancer among women living with at least one of the diseases [AOR = 2.4; 95% CI: 1.4–2.8], compared to those without these conditions. Women diagnosed with hypertension showed increased likelihood of undergoing CCS [AOR = 1.4; 95%CI: 1.1–1.7]. Similarly, women with a positive HIV test result demonstrated higher odds of screening uptake [AOR = 5.2; 95%CI: 4.0-6.7].

**Conclusion:**

The study found a positive association between comorbidities and CCS uptake in Tanzanian women. Our findings emphasize the critical importance of ensuring accessibility and adherence to essential screenings for individuals with chronic morbid conditions. Future efforts should focus on strengthening existing integrated services and identifying potential barriers to accessing CCS within these healthcare settings to optimize cervical cancer prevention efforts for individuals with chronic morbidities.

## Background

Cervical cancer remains one of the major causes of mortality worldwide. The 2019 Global Burden of Disease (GBD) shows that there were an estimated 570,000 new cases of cervical cancer, and 311,000 related deaths in 2018 [1]. Another study has shown that among the global female population aged 15 years and above who face the risk of cervical cancer, 10.2% reside in Sub-Saharan Africa (SSA) [2]. Within the SSA region, the incidence rate of cervical cancer in East Africa is 42.7 per 100,000 women [3], while in Tanzania, it is 54 per 100,000 women [4]. Extant evidence from Tanzania indicates that 9772 new cases and 6695 deaths are recorded each year [5]. Hence, making cervical cancer an important public health concern in Tanzania.

Amidst the gloomy picture of cervical cancer, there is some solace in the fact that it is a preventable and curable disease if caught and treated early. As a primary preventive measure, human papillomavirus (HPV) vaccination is recommended to prevent the development of precancerous lesions that tend to exacerbate women’s risk of cervical cancer [6]. In addition to HPV vaccination, the existing body of knowledge identifies cervical cancer screening (CCS) uptake (e.g., Pap smear test, HPV sampling test, visual methods) as a secondary preventive measure [7, 8]. Consequently, the World Health Organization (WHO) envisions that by 2030, “70% of women will be screened with a high-performance test by 35 years of age and again by 45 years of age” [8]. At the country level, the Tanzanian Ministry of Health and Social Welfare (MOHSW), in 2011, implemented VIA cervical cancer screening and cryotherapy services at more than 300 locations across the country; by 2017, the country became the seventh African country to introduce HPV vaccination in its immunization program [9]. Also, the Tanzania national strategy for cervical cancer prevention recommends that “VIA screening and cryotherapy be offered primarily at Reproductive and Child Health (RCH) clinics and that provider-initiated HIV testing and counseling (PITC) be integrated into cervical cancer screening” [10].

Despite the MOHSW’s commitment to eliminating cervical cancer in Tanzania, there is a reported low uptake of screening services (i.e., uptake prevalence of 6%) [11]. In a qualitative study by Linde et al. [12], it was found that the key barriers to CCS uptake in Tanzania included emotional factors like anxiety about the disease and apprehension regarding gynecological examinations, along with both direct and indirect economic burdens, including transportation expenses, lost income, and time spent waiting. Similarly, Mugassa et al. [13] have reported that the factors influencing women’s participation in CCS include the preference for curative over preventive services, limited information dissemination from the national to lower levels, insufficient awareness campaigns, a shortage of necessary tools and equipment, and a deficiency of skilled and proficient healthcare workers.

Beyond the identified factors, there is a growing concern about the effects of comorbidities on women’s CCS behavior [14, 15]. Comorbidity refers to the simultaneous presence of two or more diseases. Within the context of this study, it denotes the presence of hypertension and HIV. According to Constantinou et al. [15], the significance of chronic comorbidities in cancer has become imperative because *“cancer-specific mortality is higher for newly diagnosed cancer patients suffering from chronic conditions, even when stage at diagnosis or treatment are taken into account”*. HIV infection is known to suppress the immune system, thereby giving way for opportunistic infections such as HPV infection to grow. This is evident in Giles et al.’s [16] study that revealed that women with HIV are five times more likely to develop HPV infection, a known causative factor for cervical cancer. Regarding hypertension status, one qualitative study has revealed that living with hypertension deters women from undergoing CCS [17]. Another cross-sectional study [18] has found having hypertension to be associated with higher screening uptake. Thus, suggesting that there could be a possible significant association between the presence of chronic morbidities (hypertension and HIV) and women’s uptake of CCS. To date, there is no published evidence from Tanzania to show what the association is like. This demonstrates a substantial gap in what is currently known about the determinants of women’s CCS uptake in Tanzania. The aim of this study is to examine cervical cancer screening (CCS) uptake among women living with hypertension and HIV in Tanzania.

## Methods

### Data source and design

**Fig. 1:**
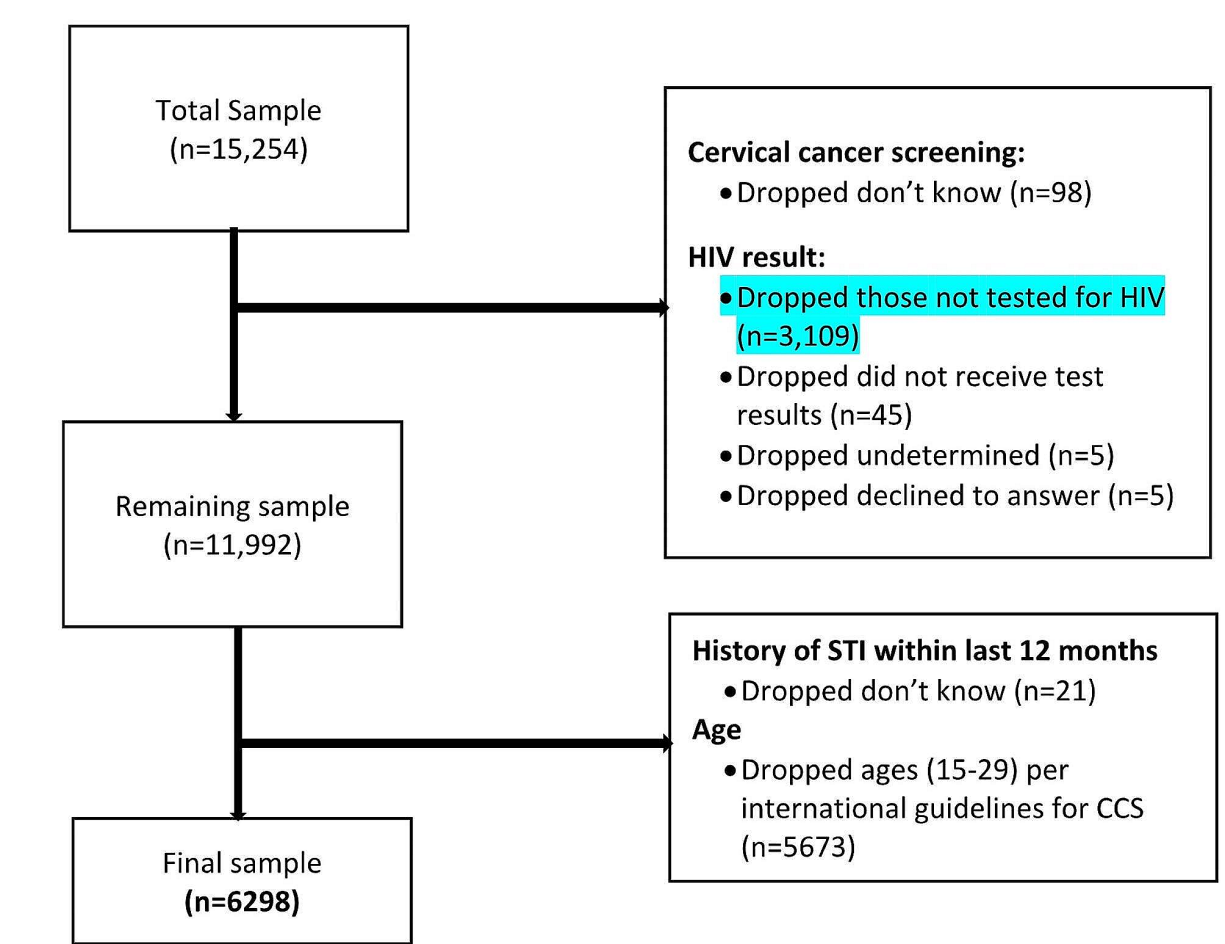
Flow chart of sampling selection

We used the recently released 2022 Tanzania Demographic and Health Survey (TDHS). This nationwide survey is carried out every five years, covering all regions of the country. The TDHS is conducted using consistent methodologies and tools, with the collaboration of the National Bureau of Statistics (NBS) and Monitoring and Evaluation to Assess and Use Results (MEASURE) DHS [19]. Furthermore, the TDHS utilizes a random sampling approach that takes population density into account to gather data from every administrative region in the country [19]. To assess regional variations in specific demographic indicators, Tanzania was segmented into nine geographic zones. While these zones are not officially recognized as administrative divisions, they are also adopted by the Reproductive and Child Health Section of the Ministry of Health [20]. Categorizing the regions into zones increased the denominator’s sample size, which, in turn, decreased the margin of sampling error [20]. For this study, we used data from the individual recode file. This file contains data from 15,154 women of reproductive age (15–49 years). The eligible age for CCS in Tanzania is 30 years [21]. For that reason, women less than age 30 were excluded from the study. Also, we excluded 3109 women who did not test for HIV at all (see Fig. 1). This resulted in a final sample of 6,298 women.

### Measures

#### Outcome variable

The outcome variable assessed in the study was cervical cancer screening (CCS). This variable was determined based on the question: “Have you ever been tested for cervical cancer by a healthcare provider?” It was coded as ‘0’ for ‘No’ and ‘1’ for ‘Yes’.

### Explanatory variables

The presence of chronic morbidities constituted the main explanatory variables. The diseases of interest were hypertension and HIV infection. Hypertensive status was self-reported data on whether the women had “ever told that they have high blood pressure”. This was coded as ‘0’ for ‘No’ and ‘1’ for ‘Yes’. HIV status was derived from the question which talks about whether the respondent had received results of their HIV test. A positive test result was coded ‘Yes’ while a negative test result was coded ‘No’.

Other explanatory variables that served as covariates in this study included history of an STI (i.e., yes or no), parity (i.e., nulliparous, uniparous, and multiparous), age (i.e., 30–34 years, 35–39 years, 40–44 years, and 45–49 years), educational level (i.e., primary, secondary, higher), wealth index (i.e., poorest, poorer, middle, richer, richest), place of residence (i.e., rural and urban), media exposure (i.e., yes or no), and the consideration of distance as a problem (i.e., big problem, not a big problem). The selection of these covariates was grounded in literature [7, 22].

### Statistical analyses

To initiate the analysis, we initially applied dataset weighting while accounting for the primary sampling unit and sampling errors. This adjustment was implemented to mitigate potential sampling bias. To account for the complex sampling design of the Tanzania DHS, the survey design was handled by utilizing the complex survey command “svyset” in STATA, which incorporates information regarding strata and primary sampling units (PSUs). This approach ensures that the estimates derived from the analysis accurately reflect the population characteristics and account for the survey’s sampling methodology. Following this data preparation, we arrived at a final sample size of 6,298, as presented in Fig. 1. To assess the association between cervical cancer screening (CCS) uptake and the main explanatory variables and covariates, we utilized a chi-square test. We employed two models for the analysis, involving both bivariable and multivariable logistic regression. For the bivariable analysis, a logistic regression model was used to estimate odds ratios to examine the associations between only chronic morbidities (hypertension and HIV) and CCS uptake. However, in the multivariable logistic regression, we adjusted for the covariates. To investigate the confounding effect of individual covariates on the association between the main independent variables (Hypertension and HIV) and the outcome of interest, the stepwise approach to model building was employed. We conducted a systematic examination of each potential confounder’s impact on the association under investigation. Initially, we assessed each covariate individually, examining its influence on the main independent variables and the outcome variable. Subsequently, we incorporated covariates with a significant level below 0.05 (*p* < 0.05) into the final Model II. All the analyses were conducted in STATA version 18 (StataCorp, College Station, TX, USA).

## Results

### Prevalence of CCS uptake across the explanatory variables

Among the participants, 614 (9.7%) and 401 (6.4%) were living with hypertension and HIV, respectively. The results show that only 975 (15.5%) of the participants were living with either one of the diseases. Out of the 6,298 respondents, only 805 (12.8%) had undergone CCS with higher screening uptake among those living with either one of the diseases (28.5%) than among those living with either hypertension or HIV. The highest proportion was found among those who had ever been diagnosed with hypertension (24.1%) and among women with positive HIV test results (36.7%). Women with a history of STI in the last 12 months reported higher prevalence of CCS uptake (15.5%). Higher proportional uptake of CCS was found among women in the age group of 45–49 years (15.2%), those with higher educational attainment (30.0%), those exposed to media (17.9%), those in the richest wealth index (22.4%), and among women who did not consider distance to the health facility as a problem (14.7%). Conversely, multiparous women (11.9%) exhibited the lowest proportions of CCS uptake, indicating potential barriers or lower utilization in these groups (see Table 1).

**Table 1:** Prevalence of CCS uptake across participants’ characteristics

Variables	Weighted sample [n (%)]	Yes, screened for cervical cancer n (%)^a^	*p*-value
**Having either one of the diseases**			< 0.001
No	5323 (84.5)	529 (9.9)	
Yes	975 (15.5)	276 (28.3)	
**Ever diagnosed of hypertension**			**< 0.001**
No	5684 (90.3)	657 (11.6)	
Yes	614 (9.7)	148 (24.1)	
**Positive HIV test result**			**< 0.001**
No	587 (93.6)	658 (11.2)	
Yes	401 (6.4)	147 (36.7)	
**History of STI within last 12 months**			**0.003**
No	5927 (94.1)	748 (12.6)	
Yes	371 (5.9)	57 (15.5)	
**Parity**			**< 0.001**
Nulliparous	194 (3.1)	39 (19.9)	
Uniparous	370 (5.9)	80 (21.5)	
Multiparous	57,374 (91.0)	687 (11.9)	
**Age**			**< 0.001**
30–34 years	1957 (31.1)	210 (10.7)	
35–39 years	1747 (27.8)	214 (12.3)	
40–44 years	1422 (22.5)	202 (14.2)	
45–49 years	1171 (18.6)	178 (15.2)	
**Place of residence**			**< 0.001**
Urban	2285 (36.3)	477 (20.9)	
Rural	4013 (63.7)	328 (8.2)	
**Educational level**			**< 0.001**
No education	1289 (20.5)	70 (5.4)	
Primary	3950 (62.7)	494 (12.5)	
Secondary	956 (15.2)	210 (21.9)	
Higher	103 (1.6)	31 (30.0)	
**Media exposure**			**< 0.001**
No	3444 (54.7)	295 (8.6)	
Yes	2854 (45.3)	510 (17.9)	
**Wealth index**			**< 0.001**
Lower	2010 (31.9)	101 (5.0)	
Middle	1243 (19.7)	102 (8.2)	
Higher	3045 (48.4)	602 (19.8)	
**Distance to health facility**			**< 0.001**
Not a big problem	4415 (70.1)	650 (14.7)	
Big problem	1883 (29.9)	155 (8.2)	
**Total**	**6298 (100.0)**	**805 (12.8)**	

^a^n(%) are row percentages

### Association between chronic morbidities and CCS uptake among women (30–49 years)

Table 2 shows the association between chronic morbidities and CCS uptake. Women diagnosed with hypertension showed increased likelihood of undergoing CCS [AOR = 1.4; 95%CI: 1.1–1.7]. Similarly, women with positive HIV test results demonstrated higher odds of screening uptake [AOR = 5.2; 95%CI: 4.0-6.7]. A higher likelihood of CCS uptake was found among women with higher educational attainment [AOR = 1.8; 95%CI: 1.4–2.4 for Primary, AOR = 2.3; 95%CI: 1.7–3.2 for Secondary, AOR = 3.3; 95%CI: 2.0-5.7 for Higher], those with a history of STI within the last 12 months increased the likelihood of CCS utilization [AOR = 1.4, 95%CI; 1.0-1.9], and those exposed to media [AOR = 1.3; 95%CI: 1.1–1.6]. Residing in rural areas was associated with lower screening uptake [AOR = 0.6; 95%CI: 0.5–0.8]. Compared to women in the lower wealth quintile, those in the upper wealth quintile were more likely to undergo screening [AOR = 2.2; 95%CI: 1.6–2.9].

**Table 2:** Association between chronic morbidities and CCS uptake

Main explanatory variables	Model I Crude Odds Ratio (COR)	Model II Adjusted Odds Ratio (AOR)
**Ever diagnosed of hypertension**		
No	Ref.	Ref.
Yes	1.9 [1.6–2.4]***	1.4 [1.1–1.7]**
**Positive HIV test result**		
No	Ref.	Ref.
Yes	4.6 [3.6–5.7]***	5.2 [4.0-6.7]***
**Educational level**		
No education	Ref.	Ref.
Primary	2.7 [2.1–3.5]***	1.8 [1.4–2.4]***
Secondary	3.9 [2.9–5.2]***	2.3 [1.7–3.2]***
Higher	6.9 [4.2–11.3]***	3.3 [2.0-5.7]***
**Age**		
30–34 years	Ref.	Ref.
35–39 years	1.1 [0.9–1.4]	1.2 [0.9–1.5]
40–44 years	1.4 [1.2–1.8]***	1.5 [1.2–1.9]***
45–49 years	1.6 [1.3-2.0]***	1.7 [1.4–2.1]***
**Place of residence**		
Urban	Ref.	Ref.
Rural	0.4 [0.3–0.4]***	0.6 [0.5–0.8]***
**History STI within last 12 months**		
No	Ref.	Ref.
Yes	1.4 [1.1–1.9]*	1.4 [1.0-1.9]*
**Media exposure**		
No	Ref.	Ref.
Yes	2.2 [1.9–2.5]***	1.3 [1.1–1.6]**
**Wealth quintile**		
Lower	Ref.	Ref.
Middle	1.6 [1.2–2.1]***	1.3 [0.9–1.7]
Upper	4.0 [3.2-5.0]***	2.2 [1.6–2.9]**
***Model Fitness***		
Prob > chi2	-	< 0.001
AIC	-	4268.241

AIC: Akaike Information Criterion; *p < 0.05, **p < 0.01, ***p < 0.001

### Association between living with either hypertension and HIV, and CCS uptake

Table 3 shows that there was a significantly higher likelihood of undergoing screening for cervical cancer among women living with at least one of the diseases [AOR = 2.4; 95% CI: 1.4–2.8], compared to those without these conditions. Similarly, higher educational attainment was associated with increased CCS utilization [AOR = 3.1; 95% CI: 1.8–5.3]. A history of STI within the last 12 months was also linked to greater odds of CCS uptake [AOR = 1.4; 95% CI: 1.0-1.9]. Additionally, women exposed to media display a higher likelihood of CCS utilization [AOR = 1.3; 95% CI: 1.1–1.5]. Conversely, residing in rural areas was associated with lower screening uptake [AOR = 0.6; 95% CI: 0.5–0.8]. The study found that women in the higher wealth quintile were more inclined to undergo screening [AOR = 1.9; 95% CI: 1.5–2.6] compared to those in the lower wealth quintile.

**Table 3:** Association between having both hypertension and HIV, and CCS uptake

Main explanatory variables	Model I Crude Odds Ratio (COR)	Model II Adjusted Odds Ratio (AOR)
**Has either one of the disease**		
No	Ref.	Ref.
Yes	2.9 [2.5–3.5]***	2.4 [1.4 − 2.8]***
**Educational level**		
No education	Ref.	Ref.
Primary	2.7 [2.1–3.5]***	1.9 [1.4–2.5]***
Secondary	3.9 [2.9–5.2]***	2.1 [1.5–2.9]***
Higher	6.9 [4.2–11.3]***	3.1 [1.8–5.3]***
**Age**		
30–34 years	Ref.	Ref.
35–39 years	1.1 [0.9–1.4]	1.2 [0.9–1.5]
40–44 years	1.4 [1.2–1.8]***	1.5 [1.2–1.8]***
45–49 years	1.6 [1.3-2.0]***	1.6 [1.3–2.1]***
**Place of residence**		
Urban	Ref.	Ref.
Rural	0.4 [0.3–0.4]***	0.6 [0.5–0.8]***
**History STI within last 12 months**		
No	Ref.	Ref.
Yes	1.4 [1.1–1.9]*	1.4 [1.0-1.9]*
**Media exposure**		
No	Ref.	Ref.
Yes	2.2 [1.9–2.5]***	1.3 [1.1–1.5]**
**Wealth quintile**		
Lower	Ref.	Ref.
Middle	1.6 [1.2–2.1]***	1.2 [0.9–1.6]
Upper	4.0 [3.2-5.0]***	1.9 [1.5–2.6]***
***Model Fitness***		
Prob > chi2	-	< 0.001
AIC	-	4340.07

AIC: Akaike Information Criterion; *p < 0.05, **p < 0.010, ***p < 0.001

## Discussion

In this study, we sought to examine the association between the presence of chronic morbidities and women of reproductive age’s uptake of CCS in Tanzania. Our findings suggest a low CCS uptake among women aged 30–49 (8.46%) in Tanzania. The observed prevalence of CCS uptake is worrying given that screening is an easy, cost-effective way to detect cervical cancer early, and initiate treatment [23, 24]. Our estimated prevalence of CCS uptake is low when compared to other Eastern African countries such as Kenya (16.4%) [18], and Uganda (20.6%) [25]. The implication is that Tanzania may not be able to achieve the WHO’s target of eliminating cervical cancer in all regions by 2030 [26]. Across the morbidities, it is observed that screening uptake was higher among those living with HIV (36.7%) than among those living with hypertension (28.5%). Individuals living with HIV often have regular contact with healthcare providers as part of their HIV management, which may facilitate greater opportunities for CCS uptake through integrated healthcare services [10]. Additionally, there may be heightened awareness and emphasis on preventive healthcare measures among individuals living with HIV due to their increased susceptibility to various health complications, including cervical cancer. On the other hand, women diagnosed with hypertension may not receive the same level of healthcare engagement specifically targeted towards preventive screenings like CCS, as hypertension management may primarily focus on blood pressure monitoring and medication adherence rather than comprehensive preventive care. However, since the estimated prevalence here is at the population level, it is possible that there would be higher uptake in specific population groups such as female sex workers [27].

Our findings support the hypothesis that the presence of chronic morbidities significantly predicts women’s likelihood to undergo screening for cervical cancer. This is evident in our findings that women living with either HIV and hypertension were 2.4 times more likely to undergo screening for cervical cancer. Specifically, women who had tested positive for HIV were five times more likely to undergo screening compared to those who had tested negative for HIV. This is consistent with Anderson et al.’s [28] study that concluded that positive HIV status plays a significant role in influencing CCS programs. It is possible that women who were HIV positive were aware of the risk of developing cervical cancer. Hence, influencing the high screening uptake. Furthermore, the findings reveal a higher screening uptake among those living with HIV compared to those with hypertension. This difference in the likelihood of screening across the two conditions may be due to Tanzania’s efforts to integrate CCS in the country’s Reproductive and Child Health (RCH) clinics and care and treatment centers (CTC) [10, 29].

Contrary to a previous study [17] that identified the presence of hypertension as a barrier to CCS uptake among women, we found that women living with hypertension were 1.4 times more likely to undergo screening. Nonetheless, our result is corroborated by a study conducted among Kenyan women [18] that found higher CCS uptake among women living with hypertension. This finding may be explained from the perspective that women living with hypertension have regular contact with healthcare providers. Therefore, it is possible that they would be exposed to information about cervical cancer, the available screening modalities, the benefits of getting screened, and the dangers of not undergoing screening. This frequent exposure to healthcare providers and health information is likely to make the woman informed and free of misperceptions and myths and allay their fears about CCS.

Consistent with existing literature [7, 22], we found higher educational attainment, increasing age, higher wealth index, and frequent exposure to the media to be associated with higher screening uptake. Women residing in rural areas were less likely to undergo screening for cervical cancer. This result aligns with similar observations reported in earlier studies by Binka et al. [30] as well as Lim and Ojo [31]. Factors such as limited access to healthcare facilities, transportation challenges, and a lack of awareness may contribute to this observed rural-urban disparity as far as screening uptake is concerned. Among the covariates analyzed, educational level exhibited the highest confounding effect. This is evidenced by the substantial decrease in odds ratios across all levels of education when moving from the unadjusted model (Model I) to the adjusted model (Model II). For instance, the odds ratios for primary, secondary, and higher education levels decreased from 2.7, 3.9, and 6.9 in Model I to 1.8, 2.3, and 3.3 in Model II, respectively. Education level is often linked to health literacy, awareness of preventive healthcare measures, and access to healthcare services [7, 22]. This may explain the strong confounding effect of education on the association between HIV and CCS uptake, and hypertension with CCS uptake.

### Strengths and limitations

Our study is the first of its kind in Tanzania to assess the independent role of chronic morbidities as a predictor of women’s CCS uptake. This contributes substantially to what is already known about the determinants of CCS uptake. Also, the TDHS follows robust sampling procedures that limit the potential for sampling biases. Again, the data used is large enough to make generalizations to the wider population of women of reproductive age in Tanzania. Nonetheless, our findings may not apply to older women (i.e., 50 years and older) who are most likely to experience more chronic morbidities. Also, the cross-sectional nature of the TDHS precludes us from establishing causality between the presence of chronic morbidities and women’s uptake of CCS. We are also unable to determine whether the chronic morbidities preceded women’s CCS uptake or vice versa. Both HIV and hypertension status were self-reported as such, there is the potential for social desirability bias. It must also be noted that the findings are only applicable at the population level. This means that it may not be applicable in high-risk populations such as female sex workers. The design of the DHS precludes us from accounting for potential temporal relationship between the onset/diagnosis of morbidity and women’s initiation of CCS. Additionally, the exclusion of women who had not tested for HIV and those aged < 30 years from the analysis may have introduced selection bias. Therefore, this should be considered when interpreting our findings.

## Conclusion

The study found a positive association between comorbidities and CCS in Tanzanian women. Our findings emphasize the critical importance of ensuring accessibility and adherence to essential screenings for individuals with chronic morbid conditions. Future efforts should focus on strengthening existing integrated services and identifying potential barriers to accessing CCS within these healthcare settings to optimize cervical cancer prevention efforts for individuals with chronic morbidities.

## Data Availability

The datasets generated and/or analysed during the current study are available in the Measure DHS repository: http://dhsprogram.com/data/available-datasets.cfm.

## Abbreviations

AIC: Akaike Information Criterion
AOR: Adjusted Odds Ratio
CCS: Cervical Cancer Screening
COR: Crude Odds Ratio
DHS: Demographic and Health Survey
TDHS: Tanzania Demographic and Health Survey
WHO: World Health Organization
